# From deception to mutualism: a continuum in *Gastrodia* brood-site pollination

**DOI:** 10.1093/jxb/erag240

**Published:** 2026-07-29

**Authors:** Oona C Lessware

**Affiliations:** Mechanical Ecology Lab, University of Exeter, Department of Biosciences, Stocker Road, Streatham Campus, Exeter EX4 4QD, UK

**Keywords:** Brood-site deception, brood-site mutualism, drosophilid pollination, floral trait evolution, *Gastrodia*, mycoheterotrophy, *Orchidaceae*

## Abstract

This article comments on:

**Suetsugu K, Hirota SK, Okui N, Okuyama Y, Kimura MT**. 2026. Drosophilid pollination in mycoheterotrophic orchids reveals a brood-site deception–mutualism continuum and phylogenetic conservatism. Journal of Experimental Botany **77**, 4679–4694. https://doi.org/10.1093/jxb/erag151

This article comments on:

**Suetsugu K, Hirota SK, Okui N, Okuyama Y, Kimura MT**. 2026. Drosophilid pollination in mycoheterotrophic orchids reveals a brood-site deception–mutualism continuum and phylogenetic conservatism. Journal of Experimental Botany **77**, 4679–4694. https://doi.org/10.1093/jxb/erag151

**Deceptive pollination represents one of the most striking examples of specialization in flowering plants. Despite extensive study of deceptive systems in orchids, evolutionary transitions between exploitation and mutualism remain poorly understood. Suetsugu *et al.* (2026) examined six drosophilid-pollinated *Gastrodia* (*Orchidaceae*) species and provide compelling evidence for an intermediate stage between deceptive and mutualistic brood-site pollination. By integrating phylogeny, pollinator identity, larval development, and ecological variation, the study shows that interactions between orchids and their pollinators may be more dynamic than previously thought. These findings highlight *Gastrodia* as a valuable system for gaining mechanistic insight into the evolution of complex floral traits.**

*Orchidaceae* (∼31 000 species) are renowned for their diversity in pollination strategies, with nearly half employing deceptive pollination (Ackerman *et al.*, 2023). Deception involves flowers presenting false reward cues. Observations date back to 1793, when Sprengel described nectarless *Orchis* (*Orchidaceae*) as ‘Scheinsaftblumen’ (‘sham-nectar flowers’), a notion initially met with scepticism from Charles Darwin (Sprengel and Sprengel, 1793; Darwin, 1877). Strategies include generalized food deception, where flowers mimic rewarding species (Jersáková *et al.*, 2006), and sexual deception, where flowers mimic female insect signals (Schiestl *et al.*, 1999). In brood-site deception, flowers use olfactory cues such as volatiles resembling rotting flesh, dung, fermenting fruit, or fungi to mimic oviposition sites (Jersáková *et al.*, 2006). This strategy occurs across angiosperms, with examples in *Rafflesia* (*Rafflesiaceae*; Beaman *et al*., 1988) and *Amorphophallus* (*Araceae*; Urru *et al*., 2011), whereas brood-site mutualism is less common. A well-documented example of brood-site mutualism is the relationship between fig trees and fig wasps, where female wasps pollinate the enclosed flowers of the fig while laying eggs inside the inflorescence, gaining a brood site for their larvae in return (Nefdt and Compton, 1996). Despite deception being common in orchids, clear intermediate stages between brood-site deception and brood-site mutualism have not been documented. Such transitional states are important for understanding how composite floral traits evolve (Kellenberger *et al.*, 2023).

In addition to plant–pollinator interactions, orchid life histories are also shaped by associations with fungi. Plant–fungi interactions are widespread in land plants (Fig. 1). Orchid seeds are small and dust-like, lacking an endosperm and nutrient reserves (Arditti and Ghani, 2000). As a result, orchids rely on colonization by fungi, which provide carbon and nutrients required for germination and early development (Rasmussen *et al.*, 2015). The orchid embryo develops into a protocorm, a transitional structure specialized for fungal colonization and nutrient uptake. In most orchids, this mycoheterotrophic phase is temporary, and seedlings transition into an autotrophic lifestyle. However, some species such as *Gastrodia* remain fully mycoheterotrophic throughout the entirety of their life cycle, having abandoned photosynthesis altogether.

**Fig. 1.**
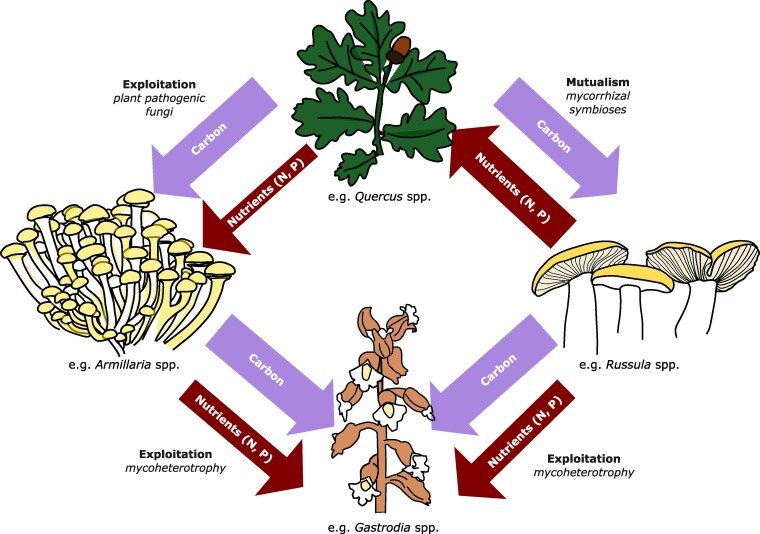
Resource exchange varies in plant–fungal interactions. Plant–fungal interactions span a continuum from mutualism to exploitation, underpinned by the net direction of carbon flow. Many interactions are mutualistic, such as in mycorrhizal symbioses, where plants gain nutrients and enhanced microbial resistance, while fungi receive fixed photosynthetic carbon essential for growth and reproduction. However, some are strongly asymmetric. Fully mycoheterotrophic plants, such as *Gastrodia*, obtain all of their fixed carbon from fungal partners (e.g. *Armillaria* spp.) and do not photosynthesize; however, this carbon is ultimately derived from photosynthetic plants via the fungal symbiont. Intermediate strategies also occur. For example, mixotrophic or partially mycoheterotrophic plants retain photosynthetic capacity while acquiring carbon from fungi. Exploitation can also occur in the opposite direction, such as in plant pathogenesis, where fungi such as *Armillaria mellea* extract resources from host plants to their detriment.

Suetsugu *et al.* (2026) present an impressive dataset spanning 15 years of field observations of six species of *Gastrodia* section *Codonanthus* (Schlechter, 1911; Tuyama, 1967) in warm-temperate and sub-tropical Japan. *Gastrodia* is the most species-rich mycoheterotrophic plant genus (>100 species). *Codonanthus* species are distributed around temperate and tropical Asia with many species in East and Southeast Asia, where they inhabit shaded forest floors. These orchids lack leaves and produce ephemeral inconspicuous flowers (Suetsugu, 2022). Many *Gastrodia* species, such as *G. similis* (Martos *et al.*, 2015), and those in sect. *Codonanthus*, are pollinated by drosophilid flies. Some species, such as *G. foetida*, employ a brood-site (nursery) pollination system, where the relationship is considered a facultative mutualism (Suetsugu, 2023). Suetsugu *et al.* (2026) combined phylogenetic reconstruction with field observations of drosophilid assemblages, larval rearing, hand pollination, and floral volatile analyses. Using this multi-pronged approach, the authors present substantial evidence that *G. confusa* and *G. pubilabiata* occupy intermediate positions between brood-site deception and brood-site mutualism.

## Drosophilid pollination in *Gastrodia*: ecology and evolutionary relatedness

Highly specialized pollination strategies, such as brood-site mutualism, and extreme niche specialization, such as mycoheterotrophy, are often associated with the radical adaptation of floral traits. When highly specialized pollination strategies evolve, shifts to different pollinators are constrained, so that evolutionary transitions tend to occur between functionally similar pollinator groups (Stebbins, 1970; Johnson and Schiestl, 2016). The field observations and phylogenetic analysis by Suetsugu *et al.* (2026) demonstrate that closely related *Gastrodia* species tend to interact with pollinators from similar ecological or functional groups (e.g. mycophagous or fruit-feeding flies).

The phylogenetic analysis reveals two well-supported clades, representing separate lineages within the sampled *Gastrodia* species. Clade 1, comprising *G. foetida* and *G. nipponica*, with *G. confusa* as a sister species to this pair; and Clade 2, comprising *G. pubilabiata* and *G. shimizuana*, with the conspicuous, white–yellow-flowered *G. gracilis* as sister species. Overlaying pollinator assemblages onto this phylogeny suggests a pattern of phylogenetic conservatism. In Clade 1, *Gastrodia* species are predominantly visited and pollinated by mycophagous drosophilids such as *Drosophila bizonata*, whilst members of Clade 2 are exclusively pollinated by fruit-feeding species such as *D. rufa*. *Gastrodia confusa*, sister to the *G. foetida/G. nipponica* clade, represents an intermediate, being pollinated by both mycophagous and fruit-feeding *Drosophila*. Drosophilid attraction is mediated primarily by olfactory cues. The researchers found that all of the three sampled representative species of *Gastrodia* emit fermentation-related volatiles, while fungi-associated volatiles are detected only in *G. foetida*.

Taken together, the results suggest that pollinator assemblages correlate with the genetic relatedness of the sampled *Gastrodia* species, providing evidence for phylogenetic conservatism of pollinator guilds across the plant species. However, this pattern may also reflect geographic distribution, habitat specificity, and spatial isolation, which can all shape pollinator assemblages independently of phylogeny. To disentangle these effects, broader sampling across *Gastrodia* is required.

## A continuum between brood-site deception and mutualism

**Fig. 2.**
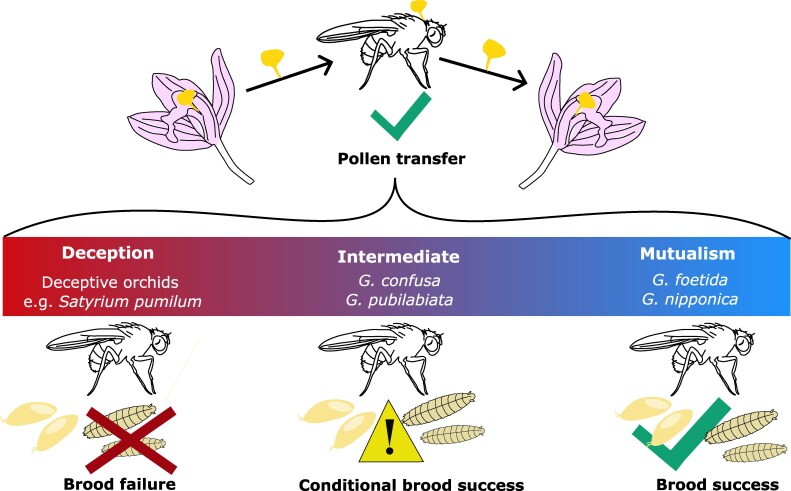
A continuum from brood-site deception to mutualism in orchid pollination. Plant–pollinator interactions are often classified as either deceptive or mutualistic. Deceptive pollination is thought to have arisen in 7500 angiosperms, two-thirds of which are orchids (Ackerman *et al*., 2023). Floral rewards are traditionally defined as nutritional resources such as nectar or pollen. However, many plant–pollinator interactions involve alternative rewards, including shelter, thermal benefits, mating opportunities, or brood sites (Jersáková *et al*., 2006). In brood-site systems, flowers provide a substrate for oviposition and larval development. As a result, the absence of nectar does not necessarily indicate deception. Suetsugu *et al*. (2026) demonstrate a gradient between these strategies in *Gastrodia* orchids. Pollination success for the plant remains consistent across the continuum, whereas benefit to the drosophilid pollinators varies. Intermediate *Gastrodia* species exhibit environmentally dependent outcomes. This challenges the binary view of deceptive versus mutualistic pollination.

A key contribution of the study is the demonstration of a brood-site deception-to-mutualism continuum. In both *G. foetida* and *G. nipponica*, drosophilid pollinators lay their eggs within the flowers, and larvae are reared feeding on the decaying floral tissues, thus providing a viable nursery for the young and indicating brood-site mutualism (Fig. 2). In the intermediate species (*G. confusa* and *G. pubilabiata*), larval success is inconsistent and dependent on environmental conditions such as humidity, rainfall, and flower detachment (Fig. 2). Historically, deceptive pollination is considered ancestral in orchids (Johnson *et al.*, 2013). If true in *Gastrodia*, then the observed gradient may suggest an evolutionary trajectory towards brood-site mutualism from an ancestrally deceptive state. Significantly, this represents the first clearly documented intermediate stage between brood-site deception and mutualism in orchids, and one of the first compelling examples of this evolutionary transitional stage in flowering plants. However, since abiotic factors and environmental conditions influence the success of larval rearing, the actual position of these species across the continuum may be more dynamic.

Analogous fragile brood-site mutualisms exist in other orchids. For example, in *Dichaea* (*Orchidaceae*), *Curculionidae* weevils act as parasitic pollinators that lay eggs in the flowers, and the larvae subsequently feed on the developing fruit. Mutualism is only achieved when parasitoid wasps reduce larval load and ‘rescue’ the flower (Nunes *et al.*, 2018). Although driven by biotic rather than abiotic factors, this similarly illustrates how brood-site mutualisms can be conditional, as in the environmentally dependent system observed in *Gastrodia*. In *Epipactis veratrifolia* (*Orchidaceae*), an interaction involving aphids and hoverflies may represent a transitional state between deception and mutualism (Jin *et al.*, 2014). However, unlike *Gastrodia*, where the continuum is defined by variation in brood-site quality within the flower itself, the *Epipactis* system involves a transition in which aphids become an indirect trophic reward for the hoverflies. Together, these three studies suggest that transitions between brood-site deception and mutualism may arise through multiple ecological and evolutionary pathways and may be more common than previously thought.

Deception is widespread in *Orchidaceae* (Ackerman *et al*., 2023), including brood-site deception where, perhaps, exploiting oviposition behaviour may be less costly than providing a true reproductive resource (Jersáková *et al*., 2006). However, empirical evidence for brood-site mutualism remains limited in orchids, and the continuum observed in *Gastrodia* suggests that these interactions may be more fluid than traditionally assumed. As a result, some systems previously classified as strictly deceptive or mutualistic through limited observations may instead occupy intermediate positions along a spectrum. Since this variation is driven by abiotic and/or biotic factors, this could be exploited experimentally to gain insight into the evolution of different pollination strategies. Controlled manipulation of climatic conditions, for example, could be used to identify thresholds at which larval development succeeds or fails, directly linking environmental conditions to brood-site success.

## Implications and future directions

The intermediate and plastic states recently documented make *Gastrodia* a promising system for mechanistic investigation of floral trait evolution, where multiple species occupying different positions along a continuum can be used to disentangle how shifts in pollination strategy and floral adaptations arise. For example, comparative analyses could test whether the position of a species along this continuum is associated with predictable shifts in floral traits or volatile emissions.

Additionally, mycoheterotrophy similarly exists along a continuum, from partially photosynthetic species to those that have completely lost the machinery for photosynthesis, relying entirely on fungal partners. Dependence on specific fungal hosts can shape the distribution, ecology, and diversification of mycoheterotrophic plants (Merckx, 2012; Kinoshita *et al.*, 2016). Due to this tight ecological dependence, mycoheterotrophs may serve as indicators of forest ecosystem integrity and are particularly vulnerable to habitat loss. Despite this vulnerability, new discoveries of highly specialized mycoheterotrophic plants continue to emerge, including recently described species such as *Thismia selangorensis* (*Thismiaceae*; Siti-Munirah *et al.*, 2025) and *Monotropa callistoma* (*Ericaceae*; Wei *et al.*, 2026). Furthermore, the increasing recognition of drosophilid flies as important pollinators in shaded forest understoreys highlights the ecological significance of small and often overlooked organisms.

Overall, this study challenges a strictly binary view of plant–pollinator interactions and instead supports the notion of a continuum of strategies within a single orchid genus. While focused on *Gastrodia* sect. *Codonanthus* in Japan, this study provides an excellent foundation for broader comparative work across *Gastrodia*, as well as orchids and mycoheterotrophic lineages more generally, opening up new avenues to explore how widespread such intermediate systems are, how they evolve, and what factors shape these dynamics.
